# Meeting Report

**DOI:** 10.1155/S1463924682000364

**Published:** 1982

**Authors:** Dan P. Manka

**Affiliations:** Independent Consultant 1109 Lancaster Avenue Pittsburgh Pennsylvania 15218 USA


Journal of Automatic Chemistry, Volume 4, Number 3 (July–September 1982), page 142–144

### Meeting Report

### The 1982 Pittsburgh Conference

More than 18 000 conferees attended the 1982 Pittsburgh Conference and Exposition on Analytical Chemistry in Atlantic City, New Jersey, USA, 8 to 12 March 1982; and 560 companies exhibited their new instruments in 1400 booths. Of the 900 papers presented at the conference, the following from the symposium on 'Process analysers' dealt with automated stream analysis for process control.

Gibbon and Hackett [1] described the use of automated research-quality gas chromatographs to analyse the effluents from bench-scale coal gasification or liquefaction equipment. The high specificity of process-control chromatographs is a disadvantage if the process gas stream being monitored is undergoing rapid and wide changes in chemical composition; this happens during process development. For example the gas composition, during an experimental run of a bench-scale coal liquefaction or gasification process development unit, may include pure nitrogen during the initial purge, unreacted feed-gas during start-up, and a variety of product compositions during the reaction phase of the experiment. Another common approach to monitoring these gases is taking 'grab' gas samples for subsequent laboratory analysis. Such an approach is labour intensive, and it is slow relative to the time scale of an on-line analysis. An alternative to this 'grab'-sampling approach is the attachment of automated research-quality gas chromatographs directly to the coal gasification or liquefaction equipment. The degree of automation of these chromatographs is defined by the analyst and may include a fully automated system that requires virtually no operator attention during routine operation. This eliminates sampling errors, eases manpower needs and time delays.

Gibbon and Hackett describe the use of Hewlett Packard 5730 gas chromatograph, with dual thermal conductivity cells, to handle routine gas analysis. Five gases, hydrogen, nitrogen, methane, carbon monoxide and carbon dioxide, can be analysed, as well as hydrocarbons (up to four carbons), hydrogen sulphide and water vapour. The columns used are (*a*) a 60/80 mesh 5A molecular sieve, 1/8 in. OD by 10 ft. stainless steel with argon as carrier gas; (*b*) an 80/100 mesh Porapak 'R', 1/8 in. OD by 12 ft. stainless steel with helium as the carrier gas. The columns and detectors are operated at 100°C, bridge currents are 120 mA (argon carrier) and 250 mA (helium carrier) and the carrier gas flow rate is 20 cc/min. Samples are injected using an air-activated Valco six-port gas-sampling valve; a 'flow type' sampling system, which is capable of sampling more than one stream. The chromatograph is calibrated daily using three quantitative standard mixtures. Response factors, based on peak areas, for each gas are adjusted every day to yield the known volume percentage concentration for each species in the standard. All samples and standards are run at a pressure of 746 mm Hg (absolute) in the injection loop.

The Hewlett Packard 3354B Laboratory Automation System is used to synchronize sample injection with its analogue to digital converters, to process the data generated from the chromatograph, and to generate an analytical report. This system monitors the feed and product gas streams from six bench-scale Fischer Tropsch reactors and from pilot plant coal gasifiers.

Manka [2] discussed the drawbacks of infra-red gas analysers. These analysers are reliable and continuous for the analysis of gases and are better than analysis of 'snap' samples by the Orsat method. However, infra-red analysers are subject to variation due to (i) gas flow; (ii) gas temperature; (iii) analyser temperature; and (iv) atmospheric pressure. These variations can be eliminated by absolute pressure regulators; Manka described this procedure in his paper. The clean process gas is heated to 50°C in a coil located in a cabinet thermostated to ±0·05°C prior to reduction in pressure to 15 psig in the first 40-E-15 regulator (Moore Products). The exit gas is then further reduced to 2 psig in the first 43-20 absolute pressure regulator (Moore Products) located in the same cabinet. The gas flows to the hydrogen thermal-conductivity cell and then to the carbon dioxide, methane and carbon monoxide infra-red analysers. The gas then flows from the last analyser to the outlet of the second 43-20 absolute pressure regulator located in the heated box. Instrument air is heated in a coil to 50°C and the pressure reduced to 15 psig in the second 40-E-15 regulator. The air flows to the inlet of the second 43-20 regulator where its pressure is reduced to 1·5 psig. The air entering the inlet of the 43-20 regulator and the stream gas entering the outlet of the regulator combine and flow to atmosphere through the side opening of the valve. Comparison of the stream pressure to the absolute vacuum built into each of the two 43-20 absolute pressure regulators maintains a constant flow and constant pressure of the process gas, regardless of changes in the original gas pressure and in atmospheric pressure. The initial gas pressure should be set at 0·1 psig to 0·2 psig above the maximum atmospheric pressure recorded at the local weather station over the previous five years.

The control of pressure by absolute pressure regulators, and the close temperature control of the regulators and analysers, has resulted in reliable analysers. Calibration of the analysers every eight hours during a 31-day test period gave less than 0·1% deviation in the gas concentration of the thermal conductivity and infra-red analysers.

If the brick and lining of a blast furnace or a heat-treating furnace is deteriorating, a small amount of a fine dust is carried into the analysers. These fine dust particles are saturated with carbon monoxide and carbon dioxide. When atmospheric air or moisture reaches this dust during a process shut-down, the two gases are released. Subsequent calibration may take as long as 1 h to reach equilibrium because the fine dust absorbs carbon monoxide and carbon dioxide from the calibrating gas. This phenomena can be eliminated by placing a normally open solenoid valve in the gas line after the last analyser, and in the gas line ahead of the inlet of the calibrating gas. When the plant process is shut-down, the solenoid valves are closed, thus trapping a gas containing carbon monoxide and carbon dioxide with the fine dust. Since the dust has not lost any carbon monoxide or carbon dioxide the subsequent calibration reaches equilibrium in the normal 1–2 min.

Process chromatographs are an integral part of a propylene concentration unit in the petroleum industry, according to Wachel and Sherman [3]. Process gas-chromatography is used at Amoco Oil Company's Whiting Refinery as an analytical input to a pneumatic-logic feed forward control system on a propylene concentration unit. This specialized chemical fractionation unit must consistently maintain stringent product specifications of not less than 99·5 weight percentage propylene, with limits on methane, ethane, ethylene, butanes and water of a few parts per million by separating a varying composition propane-propylene feed-stream. Multiple chromatographic analyses can provide information on the compositions of the tower feeds, overheads and bottoms to optimize fractionation quality, while maintaining on-specification production demand levels.

The process unit's operation must be thoroughly understood to process properly sampling, analyser selection, analyser placement and control input. The feed stock for this unit comes from the vapour recovery section of a fluid catalytic cracking unit. A de-ethanizer tower separates ethane and lighter hydrocarbons from a mixture of propane, propylene and trace butane. This serves as the feed to an efficient propylene splitter tower which accomplishes the difficult separation of propylene and propane, whose boiling points differ by only 5°C.

Exact sample take-off and return points are carefully selected to obtain a representative sample. An insertion probe $\frac{1}{4}$ in. or 3/8 in. stainless-steel tubing is used for process sampling and is located in a vertical section of pipe, thereby avoiding undissolved gases, vapours, particulates and water which may be travelling along the top or bottom of a horizontal process pipe. The sampling point is located close to the analysers to decrease sample transport times. On longer sample transport, the process sample is carried at high flow rate to the vicinity of the analyser where a slip stream is drawn off to the analyser and the remainder returned to the process downstream from the sampling point. The sample lines are steam or electric traced to maintain gaseous streams above their dew point.

The process streams are conditioned by using a series of pressure regulators, filters, and rotameters to regulate sample pressure, to remove contaminating, corrosive or harmful constituents, and to meter the flow rate before injecting into the chromatograph.

Performance validation is accomplished by equipping each analyser with stream-switching capability to analyse a primary standard.

Microprocessor-controlled process gas chromatographs with isothermal air-baths were selected to analyse the hydrocarbons from ethylene through butane, using thermal conductivity and flame ionization detectors. A combination of tower feeds, overheads, side draws and bottoms are analysed on these Applied Automation Model 2100 gas chromatographs from a few parts per million to virtually 100% weight. The chromatographs and sample conditioning systems were installed in shelters designed to maintain temperatures within the working limits of the analysers.

The three components of the gas chromatographs contain the sample conditioning system, the chromatograph oven containing the sample valve, columns and detector and the third section contains an extensive electrical section where the major advances have been made, including the addition of a computer sequencing and data manipulation capabilities. Analyser data from detectors, electronic flow-meters and alarm diagnostics are digitized and transmitted to a control unit. Each control unit can control the operating functions of four or more analysers.

The control unit can be located in an environmentally preferable location as far as a mile from the analysers, or equipped with hazardous area protective devices alongside the analysers.

The propylene concentration unit control system utilizes feed composition, feed rate and desired production rates as inputs to a feedforward/feedback control to produce desired amounts of polymer grade and chemical grade propylene. Process adjustments by the control system include product flow rates, tower reflux rates, and temperature within set point limits. Analyser signals are also accumulated and transduced to the control system per analysis cycle, and the data tabulated as historical records on a process unit computer information system for future operating and maintenance use. The finished data provided by these microprocessor-controlled gas chromatographs and the control system enables operations to produce desired quantities of high purity products, while operating optimally on closed loop control.

Conetta [4] described the analysis of organic carbon in various process streams. His system incorporates ultra-violet digestion to break down the organic material in the stream sample so that total organic material concentration can be determined. A timer introduces one of two streams, or a calibrant, into the analytical manifold. The proportioning pump moves samples and reagents through the entire system. The unit operations that follow are sparging to remove inorganic carbonate, ultra-violet digestion to breakdown the organics, gas-phase separation of the carbon dioxide formed into a buffered phenolphthalein reagent stream. The reduction of colour of the indicator is measured by the flow through a colorimeter, and the results are displayed on a strip-chart recorder. The sample is mixed with dilute sulphuric acid and the carbon dioxide formed is sparged from the analytical system by air delivered at a rate of 300 ml/min. An aliquot is resampled where it is mixed with acid persulphate or alkaline phosphate buffer if high levels of chloride are present prior to entering the ultra-violet digestor, using a 14 W ultra-violet lamp. The carbon dioxide formed as a result of the organic carbon breakdown is separated from the reaction stream by a gas permeable membrane. The carbon dioxide concentration is directly proportional to the reduction of the phenolphthalein indicator.

Comparison of results obtained by a combustion method and by the ultra-violet digestion procedure show that the recoveries with the ultra-violet technique are excellent. Total organic carbon recoveries from salt-free standards and from standards with 25% salt are virtually 100%. These are pyridine, acetic acid, 1-butanol, proline, p-nitrophenol and humic acid.

The system can also be used for the oxidation of organic phosphorus compounds. Hypophosphite is oxidized in a plating bath, which is resistant to traditional oxidizing agents—such as perchloric acid and hydrogen peroxide. Cyanide can be determined when ultra-violet digestion is used to liberate the metal-bound cyanide and separated from the sample matrix by distillation under a blanket of nitrogen. Conetta's system is currently being studied for ASTM approval. The colour reaction uses chloramin-T, pyridine, and barbituric acid.

The ultra-violet photochemical oxidation, as a general technique, is applicable to a number of parameters in a variety of sample matrices. The technique can be used in continuous-flow systems for process analysis in both off-line and on-line requirements. For example, automation of the analysis, in a process that manufactures an enzyme by fermentation, increased the efficiency of the fermentation procedure by 10% and increased the throughput.

Garber [5] suggested that manufacturers of automated analysers and microprocessors should develop more reviews of pre-analytical and post-analytical factors in large volume analyses. With the availability of microcomputers, a problem can be approached from the top and then broken down into sublevels of processes of whatever is needed—these can be handled by various integrated micro-circuits. Garber's paper dealt with the role of laboratory testing in clinics and hospitals and he pointed out those areas in the analytical process which are being handled satisfactorily and the areas which need further attention. The overall analytical process in a clinical laboratory extends from the physician's initial test order to the final review of the laboratory data by the physician, which can be minutes, hours, or days later. Regardless of how good the measurement is, or how expensive the hardware, measurement can be invalidated by a number of pre- and post-analytical factors.

Chemistry laboratory test volume in the USA has more than doubled in the last 10 years. The Americans are spending an estimated $11 billion annually on chemistry measurements; total health-care expenditure is $250 billion. Garber's laboratory handles about 18 000 samples per month. Of these 49 were unidentified by patient name, 17 had confused identification information, and four were taken from the wrong patient. These are very low percentages, but even one error of this type could have major consequences. What is needed is a universal ID system which extends back to the bedside and does not depend on human reading or transcriptions. This system uses a hand-held 'wand' reader to read the patient's ID bracelet to confirm that it is the same as on the request form. A 'universal' ID coding system is needed so that all analysers in the laboratory can read the same ID-coded label. This requires that manufacturers of analysers and microprocessors get together to settle on an acceptable universal ID-coding system, and then decide to use ID readers at the sampling station of their analysers. Thus, data storage space must be provided in the data processor of the analyser to accommodate the ID and to associate the ID with the analytical results.

Another important pre-analytical factor is the effect of time on the quality of the result reported back to the physician. This includes problems related to instability of endogenous analytes and the time relation between administration of drug dose and time of draw. Disregarding these factors can yield spuriously low drug values.

It is apparent that the microprocessor must be involved not only in the control of the reaction and measurements of analytes, but in the control of the logistics of sample handling, which centres around the ID reader. The microprocessor can then report all the information together. The clinical laboratory must have control over the details of the analysis in order to adapt the instrument to its specific needs. The analyses in the laboratory follow fairly standard methodologies.

With the development of analysers with higher throughput capacity (up to 240/h) performing 10, 20 or 30 analyses per sample, the output rate of data exceeds the capability of the technologist to review the data carefully. Garber's laboratory uses a Technicon continuous-flow sequential multiple analyser, with computer, to perform 14 test and 18 test-screening panels. Data output rate is 2700 results/h. Of this, only 350 values/h can be reviewed by the operator. Obviously, on-line computer-assisted data review is the answer. The first level of review is determined during the time of measurement by the dedicated computer. It monitors the peak shape of absorbance versus time for each of the 20 analyses. Based on predefined specifications, the instrument will accept or reject the analytical signal. Failure to meet the peak shape may be due to a short sample, improper pump action, or other hydraulic problems. The second level of data review by the dedicated computer is the determination of whether the results are within accepted operating limits or linear ranges.

Acceptable measurements of these samples have been considered to be within 2 SD of the mean value. Thus, those assays within $\pm 2$ SD of the mean are acceptable, while those beyond the $\pm 2$ SD limit are unacceptable. Under conditions of random error, this is equivalent to a 95·4% confidence interval and, thus, 5% (1 out of 20) of the observations will be unacceptable.

The need in the post-analytical factor is an on-line computer-assisted data review of these high volume analysers which should be more acceptable than the 2 SD procedure.

This is a report of just one of the many sessions held at the Pittsburgh Conference. Details of other papers can be obtained by writing to the organizers at 437 Donald Road, Department J-212, Pittsburgh, Pennsylvania 15235, USA.

**Dan P. Manka**
*Independent Consultant, 1109 Lancaster Avenue, Pittsburgh, Pennsylvania 15218, USA*

### References

1. Gibbon, G. A. and Hackett, J. P., On-line gas analysis in a process development environment (Pittsburgh Energy Technology Center, P.O. Box 10940, Pittsburgh, Pennsylvania 15236, USA).
2. Manka, D. P., Upgrading of infra-red gas analysers (Independent Consultant, 1109 Lancaster Avenue, Pittsburgh, Pennsylvania 15218, USA).
3. Wachel, L. J. and Sherman, R. E., Process chromatograph and feed forward control—a successful symbiosis (Amoco Oil Co., P.O. Box 710, Whiting, Indiana 46394, USA).
4. Conetta, A. Application of UV digestion to process effluent analysis (Technicon Industrial Systems, 511 Benedict Avenue, Tarrytown, New York 10591, USA).
5. Garber, G. C. Microprocessors in clinical laboratory instrumentation—an analytical chemist's perspective (University Hospitals and Clinics, University of Wisconsin—Madison, 600 Highland Avenue, Madison, Wisconsin 53792, USA).

